# Effect of Al_2_O_3_ and ZrO_2_ Filler Material on the Microstructural, Thermal and Dielectric Properties of Borosilicate Glass-Ceramics

**DOI:** 10.3390/mi14030595

**Published:** 2023-03-02

**Authors:** Dilara Arıbuğa, Oğuz Karaahmet, Özge Balcı-Çağıran, Buğra Çiçek

**Affiliations:** 1 Graduate School of Sciences and Engineering, Koç University, 34450 Istanbul, Turkey; 2 Koç University Boron and Advanced Materials Application and Research Center, 34450 Istanbul, Turkey; 3 Akcoat R&D Center, 2nd IZ, 54300 Sakarya, Turkey; 4 Department of Metallurgical and Materials Engineering, Yıldız Technical University, 34210 Istanbul, Turkey; 5 Department of Chemistry, Koç University, 34450 Istanbul, Turkey

**Keywords:** glass-ceramics, Al_2_O_3_ filler material, ZrO_2_ filler material, microstructure, thermal conductivity, dielectric constant

## Abstract

Various glass-ceramics are widely used or considered for use as components of microelectronic materials due to their promising properties. In this study, borosilicate glass was prepared using the powder metallurgical route and then mixed with different amounts of Al_2_O_3_ and ZrO_2_ filler materials. Glass-ceramics are produced by high-energy ball milling and conventional sintering process under Ar or air. In this study, the effects of different filler materials and different atmospheres on the microstructural, thermal and dielectric properties were investigated. The data showed that ZrO_2_ filler material led to better results than Al_2_O_3_ under identical working conditions and similar composite structures. ZrO_2_ filler material significantly enhanced the densification process of glass-ceramics (100% relative density) and led to a thermal conductivity of 2.904 W/K.m, a dielectric constant of 3.97 (at 5 MHz) and a dielectric loss of 0.0340 (at 5 MHz) for the glass with 30 wt.% ZrO_2_ sample. This paper suggests that prepared borosilicate glass-ceramics have strong sinterability, high thermal conductivity, and low dielectric constants, making them promising candidates for microelectronic devices.

## 1. Introduction

Low-temperature co-fired ceramics (LTCCs) represent one of the most important technologies due to growing interest in miniaturized and integrated devices. LTCCs are multilayered ceramic materials that are notable for their remarkable physical and chemical properties [1,2,3,4,5,6]. LTCC technology is also used to make antennas, telescope mirror blanks, and sensors. It is also widely used in automotive and mobile technologies, as well as in the military [7,8,9]. The high rates of productivity in the wireless communication industries, which are increasingly inclined toward miniaturization and integration, are the main causes of the scientific and industrial community’s recent preoccupation with LTCC technology [10]. However, metal electrodes must be chemically compatible with the LTCC composites, and their sintering temperature must be lower than the melting points of highly conductive electrodes such as Ag or Au [11,12,13,14,15,16].

Glass-ceramics can be developed and optimized for their porosity, hardness, strength, opacity, thermal expansion, temperature stability, thermal conductivity, and resistivity. Particularly, ceramic substrates with glass content embedded in them offer multifunctional properties including high strength and high durability, which play a significant role in various exciting applications [17,18]. Owing to such optimizable properties, glass-ceramics are frequently utilized in industry and technological fields such as thermal-shock-resistant glass containers, fiberglass, high-level nuclear waste glass, and liquid crystal display substrates [19]. Glass-ceramics can be used as dielectric materials for electrical and electronic applications. These applications, however, are not cost-effective, have certain conductivity issues, and need high sintering temperatures [17,20] because the dielectric properties of glass-ceramics are determined by porosity, chemical structure, and crystallinity.

Several glass-ceramic systems have been developed for LTCC applications. Among several glass applications, including LTCCs, using boron oxides such as boron trioxide in glass/alumina composites is a new method that has been mentioned previously [21,22]. B_2_O_3_ addition to the B_2_O_3_—Al_2_O_3_—SiO_2_ glass-ceramic systems provides increased bending strength. Luo et al. [22] studied the effect of Al_2_O_3_ on densification, dielectric constant, dielectric loss, thermal expansion, and flexural strength in borosilicate glass. The densification and properties of glass-ceramics were improved with the addition of 4 wt.% Al_2_O_3_. Adding fillers is an effective method to synthesize solid materials with higher thermal conductivity [21,23]. The addition of BN and PVA to a low-alkali borosilicate glass-ceramic was investigated for its effect of sinterability, thermal conductivity and dielectric constant in a previous study [21]. The thermal conductivity and dielectric constants increased with 12 wt.% BN addition with a nearly fully dense structure. Additionally, the PVA provided an increase in the thermal conductivity of glass-ceramics. The use of fillers in LTCCs to improve thermal conductivity is still under investigation. Sintering additives can also be introduced to enhance the thermal conductivity along with an enhancement in the densification procedure [24]. As a result, selecting additives is as crucial as choosing glass-ceramic powders. Few studies have compared the impact of Al_2_O_3_ and ZrO_2_ as additives on the sintering process and the thermal and dielectric properties of glass-ceramics [25].

In the present study, borosilicate glass particles containing oxides such as SiO_2_, B_2_O_3_, and Al_2_O_3_ were used to research glass-ceramic materials [26]. This article investigated the effects of Al_2_O_3_ and ZrO_2_ additives on the microstructure, sinterability, thermal conductivity, and dielectric characteristics of a borosilicate glass sample. Borosilicate glass particles were combined with Al_2_O_3_ or ZrO_2_ additives and sintered at temperatures below 950°C under various atmospheres. Because of its unique traits such as low dielectric constant, strong mechanical strength, thermal properties, and chemical stability [27], borosilicate glass was chosen. The combination of Al_2_O_3_ and SiO_2_ improves the mechanical and chemical properties; however, at higher temperatures, the mechanical and chemical properties deteriorate [27]. B_2_O_3_ was utilized in the formulation to avoid this issue [21,28]. Apart from the conventional melt-quenching method, a new powder metallurgical route was used to prepare the glass powder. Finally, the features and future prospects of the investigated glass-ceramic materials in electronic systems with low-temperature sintering, high thermal conductivity, and low dielectric constant will be discussed in this article.

## 2. Experimental Methods

### 2.1. Preparation of Powders and Sintered Ceramics

Glass with the chemical composition of SiO_2_ (75 wt.%) (99% purity, 75 μm particle size), B_2_O_3_ (20 wt.%) (˃98% purity, ~15 µm particle size), and Al_2_O_3_ (5 wt.%) (99% purity, 75 μm particle size) was prepared through a powder metallurgical route, including the mechanical-milling-assisted annealing technique. High-purity (>99%) starting materials were mechanically activated using high-energy ball milling (HEBM). Milling experiments were performed using a Spex 8000D Mixer Mill equipped with hardened steel balls and vials. The powders were milled at room temperature for 3 h using a ball-to-powder mass ratio (BPR) of 10:1 and at a milling speed of 1200 rpm. Then, they were placed in a tube furnace at 950 °C for 6 h and then rapidly cooled to room temperature to obtain a suitable amorphous structure of the glass phase. The heating temperature was determined after several trials, starting from 750 °C and remaining at 950 °C as crystallization occurred above this temperature. The obtained glass was mixed with different filler materials, i.e., aluminum oxide (Al_2_O_3_) and zirconium oxide (ZrO_2_), by HEBM. The glass composites containing 30, 40, and 50 wt.% of Al_2_O_3_ or ZrO_2_ filler material were uniaxially pressed at 300 MPa to form pellets with a diameter of 10 mm and a height of 2–2.5 mm. For comparison purposes, the glass composites with filler additions were sintered at 950 °C for 1 h under Ar (2 L/min) or air atmosphere. Ar sintering was performed after sealing the working atmosphere and flowing high-purity (99.999%) Ar gas during the whole process. For air sintering, on the other hand, experiments were performed under atmospheric conditions. Glass synthesis and sintering experiments were carried out in a Protherm PTF series tube furnace. The glass composites containing 30, 40, and 50 wt.% of Al_2_O_3_ sintered under Ar and air atmosphere are hereafter termed as 30Al-Ar, 40Al-Ar, 50Al-Ar, 30Al-O, 40Al-O, and 50Al-O, respectively. Similarly, the glass composites containing 30, 40, and 50 wt.% of ZrO_2_ sintered under Ar and air atmosphere are hereafter termed as 30Zr-Ar, 40Zr-Ar, 50Zr-Ar, 30Zr-O, 40Zr-O, and 50Zr-O, respectively. Table 1 summarizes the sample names and their production conditions.

### 2.2. Characterizations

The phase structure of the samples was investigated using a Rigaku Miniflex X-ray diffractometer (XRD, CuK_α_) and the powder diffraction file (PDF) database. For microstructural investigations, samples were subjected to a conventional metallographic preparation method. After being cold-mounted with epoxy glue, specimens were ground and polished in a Tegramin 30 Struers instrument. The polished samples were examined microstructurally and morphologically using a Zeiss Ultra Plus field-emission scanning electron microscope (FE-SEM) equipped with an energy-dispersive X-ray spectrometer (EDS). The SEM images were obtained using a secondary electron (SE) detector with an acceleration voltage of 8 kV and a working distance of 8 mm. For EDS measurements and EDX mapping, a Bruker XFlash 5010 EDS detector with a resolution of 123 eV was used. An optical microscope was also used to check the samples’ quality (SOIF). Archimedes’ method was used to determine the average density values of the sintered products in ethanol by taking three iterative measurements for each sample. The average experimental density (obtained using the Archimedes method) is divided by the bulk density of the glass-ceramic composites that are created (calculated according to the filler material content) and multiplied by 100 to find the relative density (%). The density of glass powder is measured as 2.25 g/cm^3^. Following that, the average density is the density estimated by dividing the mass by the volume of the prepared samples. The thermal diffusivity of the disc-shaped cylindrical pellets was measured at room temperature. NETZSCH LFA HT467 laser flash apparatus was used to perform the thermal diffusivity measurements, denoted as D. The thermal conductivity of the samples was determined using the formula *κ = DC_P_d*, where C_P_ is the theoretical heat capacity of SiO_2_, and d is the density of the samples. Using a Hioki IM3570 impedance analyzer and the parallel plate method, the dielectric characteristics at 5 MHz were examined. A Quorum Sputter Coater was used to coat the parallel surfaces of the samples with conductive gold–palladium alloy. A sinusoidal voltage was supplied to polarize the sample to create an alternating electric field. Equation (1) was used to derive the dielectric constant from the capacitance measurements at room temperature, where *K* is the dielectric constant, C is the capacitance, d is the sample thickness, Ɛ is the permittivity of vacuum, and A is the sample area.

(1) $$K=\frac{C\cdot d}{\varepsilon \cdot A}$$

## 3. Results and Discussion

**Figure 1:**
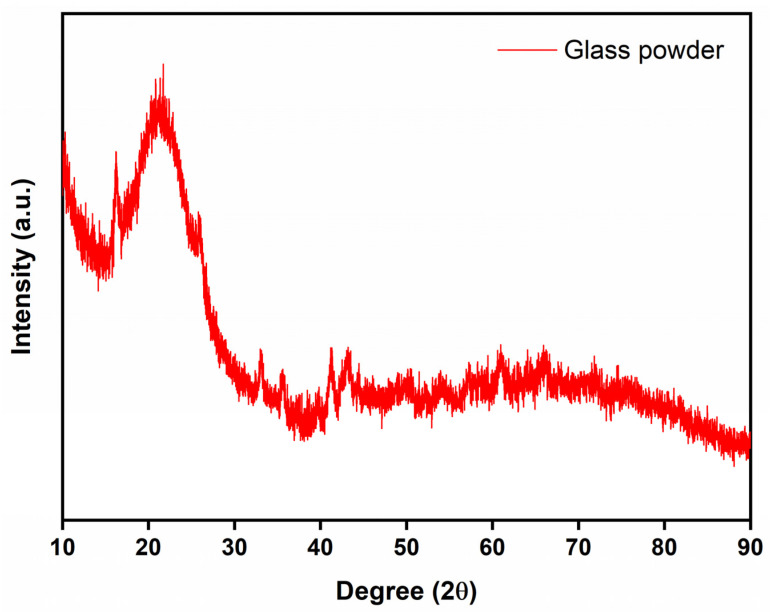
XRD pattern of the prepared glass powder.

**Figure 2:**
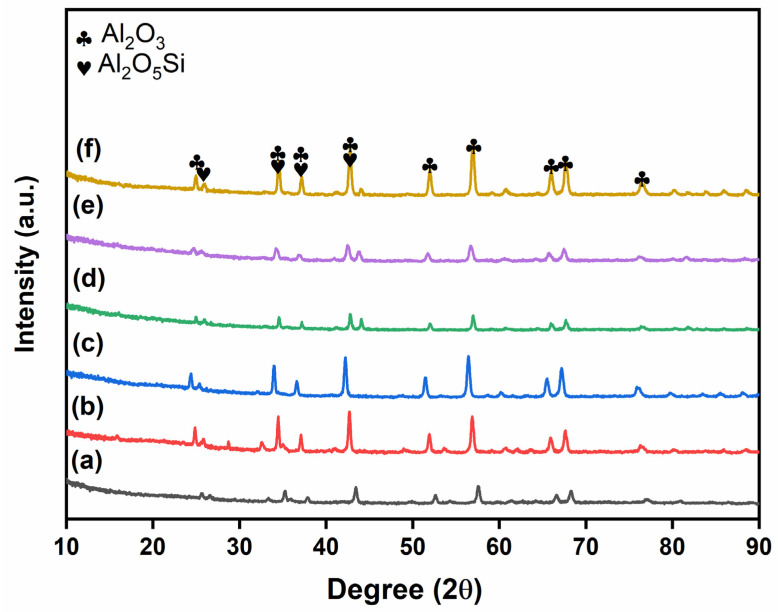
XRD patterns of glass-ceramics with Al_2_O_3_ additive: (a) 30Al-O, (b) 40Al-O, (c) 50Al-O, (d) 30Al-Ar, (e) 40Al-Ar, and (f) 50Al-Ar.

**Figure 3:**
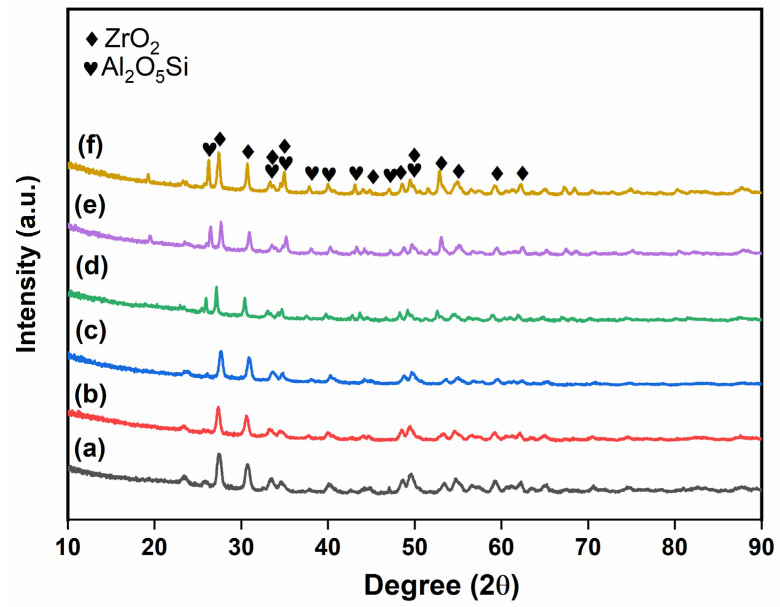
XRD patterns of the glass-ceramics with ZrO_2_ additive: (a) 30Zr-O, (b) 40Zr-O, (c) 50Zr-O, (d) 30Zr-Ar, (e) 40Zr-Ar, and (f) 50Zr-Ar.

The XRD patterns of prepared glass powder and glass-ceramics with additives sintered at 950 °C are shown in Figure 1, Figure 2 and Figure 3. The obtained phases are listed in Table S1 (in the Supplementary Information file), along with their respective PDF card numbers and crystal structures. The XRD analysis of the prepared glass powders showed that borosilicate glass powders were obtained in amorphous structures, and no crystallization was observed at the synthesis temperature (Figure 1). After sintering with filler materials, crystallization occurred in the obtained glass-ceramics. Figure 2 shows the XRD patterns of glass-ceramics with Al_2_O_3_ additive sintered under Ar and air. The crystallinity of glass-ceramics with Al_2_O_3_ addition increased as the additive amount increased from 30 to 50 wt.% (Figure 2). Additionally, when the sintering atmosphere was altered from Ar to air atmosphere, the crystallinity of the sample did not change. The XRD patterns of all the obtained powders clearly show the Al_2_O_3_ and Al_2_O_5_Si phases (PDF Card No: 9006525). On the other hand, ZrO_2_ addition has a different effect on the glass-ceramics. The obtained phases change as the sintering atmosphere changes. Figure 3 shows the XRD patterns of glass-ceramics with ZrO_2_ additive sintered under Ar and air and indicates the ZrO_2_ and Al_2_O_5_Si (PDF Card No: 9000713) phases. When the content of ZrO_2_ in glass-ceramics is raised, the crystallinity decreases, unlike when the quantity of Al_2_O_3_ is increased. This is most likely due to the high hardness of ZrO_2_ powders, which affected the powders more than Al_2_O_3_ during the milling process. The mechanical milling process decreased the crystallite size of powders and hence decreased the peak intensity of the phases after sintering.

Table 2 shows the density values of the sintered glass-ceramics. According to these measurements, glass–Al_2_O_3_ composites had a lower density than those of glass–ZrO_2_ composites. Glass–Al_2_O_3_ composites sintered under Ar or air showed lower densification than 80%, but the glass–ZrO_2_ composites sintered under air showed high bulk density values (˃90%). In contrast to glass–Al_2_O_3_ composites, adding 30–40 wt.% ZrO_2_ under Ar results in full densification (100%). Similarly, samples with ZrO_2_ addition sintered under air resulted in almost full densification results of 90–94%. It is obvious that the sintering of glass–ZrO_2_ composites under Ar significantly increased the densification of the samples. Sinterability improves when crystallite size of the powder is reduced [29]. Therefore, the addition of ZrO_2_ could increase the densification of the samples [30]. In the literature, the addition of Al_2_O_3_ and ZrO_2_ increases the densification of Li_2_O—ZrO_2_—SiO_2_—Al_2_O_3_ (LZSA) glass powder at higher temperatures. The densification ratio of ZrO_2_-added LZSA glass was higher than that of Al_2_O_3_-added LZSA glass when sintering was performed at 1000 °C [31]. Similar results were obtained in this study.

**Figure 4:**
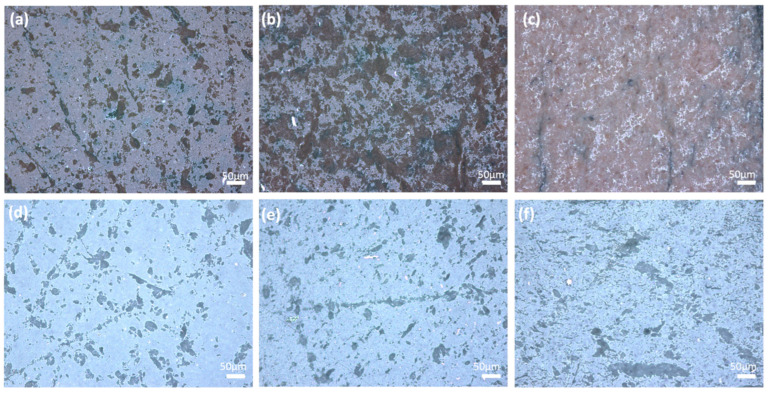
Optical microscope images of the sintered ceramics: (**a**) 30Al-O, (**b**) 40Al-O, (**c**) 50Al-O, (**d**) 30Zr-Ar, (**e**) 40Zr-Ar, and (**f**) 50Zr-Ar.

The optical microscope images of the sintered glass-ceramics are shown in Figure 4. Glass–Al_2_O_3_ composites sintered under Ar are shown in Figure 4a–c, whereas glass–ZrO_2_ composites sintered under air are shown in Figure 4d–f. The number of closed pores increased as the amount of Al_2_O_3_ increased, as illustrated in Figure 4a,b; when the amount of ZrO_2_ was decreased from 40 wt.% to 30 wt.%, the number of closed pores decreased (Figure 4e,f). It has already been reported that having a large number of closed pores is harmful to the thermal conductivity of the material and hence the performance of the sintered products [32]. Closed porosity is seen in the microstructure of the 30Al-O and 40Al-O samples, as shown in Figure 4a,b. ZrO_2_ affects the low number of large closed pores positively, as seen in Figure 4d,e; however, there are still small closed pores throughout the sample.

**Figure 5:**
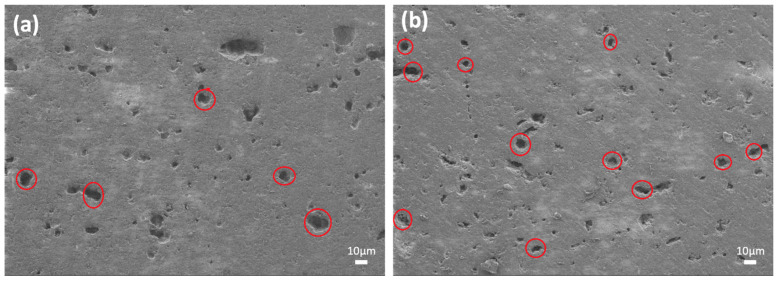
SEM images of the sintered glass-ceramics at 1.00 KX: (**a**) 30Al-O and (**b**) 40Al-O.

**Figure 6:**
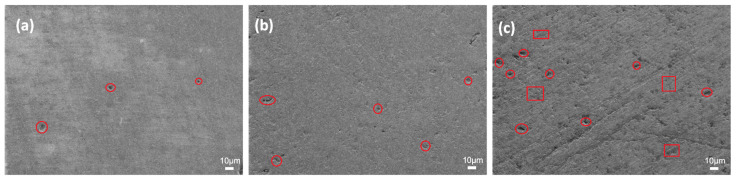
SEM images of the sintered glass-ceramics at 1.00 KX: (**a**) 30Zr-Ar, (**b**) 40Zr-Ar, and (**c**) 50Zr-Ar.

FE-SEM was performed on the samples to investigate the microstructures in detail. Figure 5 and Figure 6 display SEM images of the sintered ceramics with Al_2_O_3_ and ZrO_2_ additions, respectively. The SEM image of the glass–Al_2_O_3_ composites reveals the open pores randomly agglomerated in some areas of the sample, with some of them marked with a circle. As shown in Figure 5a, the 30Al-O sample had mainly fewer open pores. The SEM images of the 40Al-O sample showed a similar distributed pattern of the pores (Figure 5b). These areas, however, did not reflect the entire sample, which contains micron-scale closed holes throughout the microstructure.

Closed and open (sizes up to 5 µm) pores are observed in the microstructures of the glass-ceramics sintered with ZrO_2_ addition, as shown in Figure 6. Further, 30 and 40 wt.% ZrO_2_ additions significantly decreased both closed and open pores. As a result, the SEM images in Figure 6a,b showed complete densification with very few pores, showing that the sinterability of the glass-ceramic material has improved. According to the density measurements in Table 2, this structure could reduce pore development and hence increase densification. Figure 6c shows a homogeneous microstructure of the glass-ZrO_2_ composite sample with the highest ZrO_2_ content. However, a significant number of visible pores may be seen after adding 50 wt.% ZrO_2_, which is compatible with relatively low-density data (Table 2). On the other hand, the samples with the lowest ZrO_2_ content (Figure 6) produced the lowest number of pores. This phenomenon is related to the amount of ZrO_2_ filler material: When the ZrO_2_ amount is higher than 40 wt.%, the sinterability of glass-ceramics is affected negatively, and extra closed pores are created in the microstructure. The open pores are marked with a circle, whereas the closed pores are marked in a rectangular area in Figure 6. The SEM images conform well with the density measurements in Table 2, where 100 % density was obtained for the 30Zr-Ar and 40Zr-Ar samples. The high-magnification SEM images of the samples are presented in Figures S1 and S2 in the Supporting Information file.

**Figure 7:**
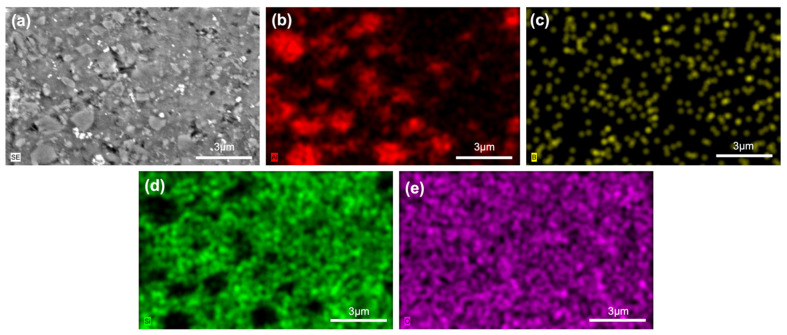
SEM/EDX analyses of the 30Al-O sample: (**a**) SEM image, (**b**) EDX mapping of Al, (**c**) EDX mapping of B, (**d**) EDX mapping of Si, and (**e**) EDX mapping of O.

**Figure 8:**
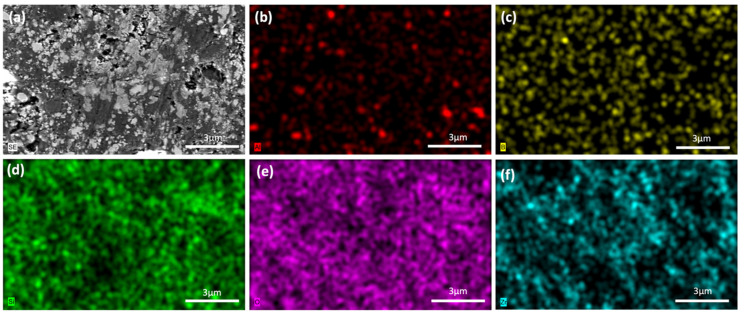
SEM/EDX analyses of the 40Zr-Ar sample: (**a**) SEM image, (**b**) EDX mapping of Al, (**c**) EDX mapping of B, (**d**) EDX mapping of Si, (**e**) EDX mapping of O, and (**f**) EDX mapping of Zr.

The 30Al-O, 40Al-O, 30Zr-Ar, and 40Zr-Ar samples were subjected to EDS analysis. Figure 7 and Figure 8 show the SEM/EDX analyses of the selected samples, while Figures S3–S8 (in the Supporting Information file) present the analyses of the other samples. All measurements of the composites with Al_2_O_3_ additive yielded the elements Al, Si, B, and O. The homogenous distributions of the Al, Si, B, and O elements are visible in the glass–Al_2_O_3_ composite of 30Al-O (Figure 7). The primary difficulties seen in conventional glass-ceramic processes, such as a lack of well-distributed particles, the emergence of clustered areas, or the creation of secondary phases, were not detected in the prepared samples, according to EDX studies. This could be related to the preparation procedure of the composites, which includes room-temperature mechanical milling followed by low-temperature sintering. The XRD patterns in Figure 1, Figure 2 and Figure 3 are consistent with these findings. In the 40Zr-Ar sample, the ZrO_2_ phase was discovered. The homogenous distributions of the elements Zr, Al, Si, B, and O can be seen in all microstructures. The Zr element signals, which are likewise uniformly distributed, are also produced by the glass–ZrO_2_ composites.

**Figure 9:**
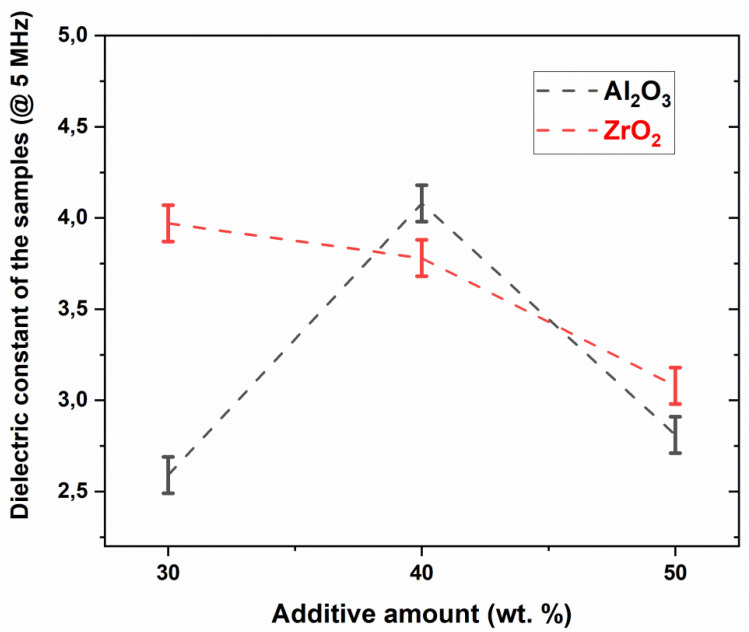
Dielectric constant of the samples according to the varying amounts of Al_2_O_3_ and ZrO_2_ additives.

Figure 9 shows the dielectric constant of the samples according to the varying amounts of Al_2_O_3_ and ZrO_2_ additives. The dielectric constants were determined to be between 2.59 and 4.08 at 5 MHz. The dielectric constant of the samples increased from 2.59 to 4.08 as the Al_2_O_3_ content increased from 30 to 40 wt.%, and over 40 wt.% of Al_2_O_3_ content resulted in a drop in the values. The sample with 30 wt.% ZrO_2_ additive had a dielectric constant of 3.97. The increase in the ZrO_2_ amount from 30 to 50 wt.% gradually decreased the dielectric constant, as can be clearly seen in Figure 9. According to the dielectric measurements, the samples 30Al-O, 40Al-O, 50Al-O, 30Zr-Ar, 40Zr-Ar, and 50Zr-Ar led to the dielectric loss values (at 5 MHz) of 0.0984, 0.2581, 0.2781, 0.0340, 0.2660, and 0.4710, respectively. The lowest dielectric loss among the samples was obtained as 0.0340 for the 30Zr-O sample. Therefore, the variation in the dielectric constant due to the amount of Al_2_O_3_ in the samples is attributed to the presence of abundant defects in the microstructure of ceramics, such as porosity [33].

Table 3 displays the measured thermal diffusivity values of the samples and their standard deviations and computed thermal conductivity values. The experimental relative density values in Table 2 were used to calculate thermal conductivity. The theoretical heat capacity was set, and the density was set to the experimental relative density values in Table 2. Glass-ceramics sintered with Al_2_O_3_ yielded lower values than those sintered with ZrO_2_, as shown in Table 3. The thermal conductivity of glass-ceramics ranges from 2.869 to 2.904 W/K.m when ZrO_2_ is used as a filler material in the composites. The thermal conductivity ranges from 1.336 to 1.507 W/K.m when Al_2_O_3_ is used as a filler material in the composites. Enhanced thermal conductivity was found despite the presence of closed pores in the microstructures of the 30Zr-Ar sample. This is most likely due to the porous structure (smaller and more evenly distributed) and the superior densification process, highlighting the importance of the sintering environment and additives chosen. The experimental and theoretical studies revealed that pore size, in particular, has a significant impact on the thermal properties of the products [26].

Furthermore, glass-ceramics’ excellent heat conductivity and their composites contribute to this result since no Al_2_O_3_ or ZrO_2_ degradation was detected due to the sintering temperature and preparation techniques used. According to the previous literature, AlN, Si_3_N_4_, SiC, and BN have also been used instead of Al_2_O_3_ and ZrO_2_ [15,21,23,34]. However, glass-ceramics with ZrO_2_ additives show a more effective way to contribute to composites than Al_2_O_3_ by comparing their thermal conductivity values with different glass-ceramic content [35]. In this study, overall, the composites with ZrO_2_ addition provided higher thermal conductivities than those with Al_2_O_3_ addition when the additive amount was set at 30 and 40 wt.%. 

The 30Zr-Ar sample, which has full densification (Table 2), a high thermal conductivity of 2.904 W/K.m (Table 3), and a dielectric constant of 3.78 (Figure 9), was found to have the best results. Therefore, it is indicated that the ZrO_2_ filler material effectively optimized microstructure and density, as well as dielectric and thermal properties. Overall, the findings are compared to commercially made LTCC glass-ceramics using B_2_O_3_ to lower the sintering temperature. It was discovered that their thermal conductivity values (2.0–4.9 W/m.K) and the dielectric constant values (2.40–4.43 MHz) were comparable to those in the current study. It is worth noting that commercially produced materials with similar thermal and dielectric properties were sintered at a lower temperature than glass-ceramics. Furthermore, the additions of ZrO_2_ and Al_2_O_3_ filler materials appear to affect the thermal and dielectric properties of the resulting glass-ceramics. This paper suggests that borosilicate glass-ceramics with additives can be good candidates for LTCC applications.

## 4. Conclusions 

In this study, we prepared glass-ceramic materials based on the borosilicate glass, glass–Al_2_O_3_, and glass–ZrO_2_ composites through the mechanical-milling-assisted annealing technique. Utilizing the glass composition and additions, the sintering temperature decreased to 950 °C, and nearly fully or fully densified samples were obtained. The thermal conductivity values were calculated as between 1.336 and 2.904 W/K.m, whereas the dielectric constant values were obtained as between 2.59 and 4.08 for all the samples. Results showed that ZrO_2_ filler material led to better results than Al_2_O_3_ under identical working conditions and similar composite structures. ZrO_2_ filler material significantly enhanced the densification process of glass-ceramics (full densification with few closed/open pores in the microstructure) and led to a thermal conductivity value of 2.904 W/K.m and a dielectric constant of 3.97 (at 5 MHz) for the glass 30 wt.% ZrO_2_ sample. The generated borosilicate glass-ceramic composites have strong sinterability, high thermal conductivity, and low dielectric constant, making them promising candidates for microelectronic devices. Furthermore, the samples’ low sintering temperature of 950 °C opens new possibilities in LTCC applications.

## Figures and Tables

**Table 1:** Sample names and their production conditions.

Sample Name	wt.% of Glass Powder	wt.% of Al_2_O_3_ Filler	wt.% of ZrO_2_ Filler	Sintering Condition
30Al-Ar	70	30	-	950 °C, Ar
40Al-Ar	60	40	-	950 °C, Ar
50Al-Ar	50	50	-	950 °C, Ar
30Al-O	70	30	-	950 °C, air
40Al-O	60	40	-	950 °C, air
50Al-O	50	50	-	950 °C, air
30Zr-Ar	70	-	30	950 °C, Ar
40Zr-Ar	60	-	40	950 °C, Ar
50Zr-Ar	50	-	50	950 °C, Ar
30Zr-O	70	-	30	950 °C, air
40Zr-O	60	-	40	950 °C, air
50Zr-O	50	-	50	950 °C, air

**Table 2:** Density results of the glass-ceramics.

Sample Name	Average Density (g/cm^3^)	Relative Density (%)
30Al-Ar	2.399	75.99
40Al-Ar	2.665	72.86
50Al-Ar	2.710	62.74
30Al-O	2.435	77.11
40Al-O	2.581	70.77
50Al-O	2.799	64.80
30Zr-Ar	2.748	100.00
40Zr-Ar	2.967	100.00
50Zr-Ar	3.012	93.48
30Zr-O	2.500	90.99
40Zr-O	2.761	93.07
50Zr-O	3.022	93.76

**Table 3:** Thermal conductivity of the samples.

Sample Name	Diffusivity (mm^2^/s)	Standard Deviation (mm^2^/s)	Average Thermal Conductivity (W/K.m)
30Al-O	0.443	0.102	1.336
40Al-O	0.472	0.017	1.507
30Zr-Ar	0.992	0.133	2.904
40Zr-Ar	0.976	0.138	2.869

## Supplementary Materials

The following supporting information can be downloaded at: https://www.mdpi.com/article/10.3390/mi14030595/s1, Figure S1: SEM images of sintered glass ceramics at 20.00 KX (a) 30Al-O, (b) 40Al-O, (c)50Al-O; Figure S2: SEM images of sintered glass ceramics at 20.00 KX (a) 30Zr-Ar, (b) 40Zr-Ar, (c) 50Zr-Ar; Figure S3: EDS analysis of the 30Al-O sample; Figure S4: EDS analysis of the 40Al-O sample; Figure S5: EDS analysis of the 30Zr-Ar sample; Figure S6: EDS analysis of the 40Zr-Ar sample; Figure S7: SEM/EDX analyses of the 40Al-O sample (a) SEM image, (b) EDX mapping of Al, (c) EDX mapping of B, (d) EDX mapping of Si, (e) EDX mapping of O; Figure S8: SEM/EDX analyses of the 30Zr-Ar sample (a) SEM image, (b) EDX mapping of Al, (c) EDX mapping of B, (d) EDX mapping of Si, (e) EDX mapping of O, (f) EDX mapping of Zr; Table S1: PDF card numbers and their corresponding crystal structures.
