# Supplementary material for: Effect of Al2O3 and ZrO2 Filler Material on the Microstructural, Thermal and Dielectric Properties of Borosilicate Glass-Ceramics
Source: Micromachines (Basel). 2023 Mar 2;14(3):595. doi: 10.3390/mi14030595 (PMC10051759; doi:10.3390/mi14030595)
Supplement: Supplementary file 1 [file micromachines-14-00595-s001.zip › micromachines-2096437-SI.pdf]

micromachines

Supplementary Materials: Effect of Al_2O_3 and ZrO_2 Filler Material on the Microstructural, Thermal and Dielectric Properties of Borosilicate Glass-Ceramics

Dilara Arıbuğa [1,2], Oğuz Karaahmet [3,4] Özge Balcı-Çağıran [2,5] and Buğra Çiçek [4,*]

1 Graduate School of Sciences and Engineering, Koç University, 34450 Istanbul, Turkey
2 Koç University Boron and Advanced Materials Application and Research Center, 34450 Istanbul, Turkey
3 Akcoat R&D Center, 2nd IZ, 54300 Sakarya, Turkey
4 Department of Metallurgical and Materials Engineering, Yıldız Technical University, 34210 Istanbul, Turkey
5 Department of Chemistry, Koç University, 34450 Istanbul, Turkey
* Correspondence: bcicek@yildiz.edu.tr

Table S1. PDF card numbers and their corresponding crystal structures.

PDF card number	Chemical formula	Mineral name	Crystal structure
**9007635**	Al_2O_3	Corundum	Hexagonal
**9006525**	Al_2O_5Si	Silimanite	Orthorhombic
**8104264**	ZrO_2	-	Monoclinic
**9000713**	Al_2O_5Si	Silimanite	Orthorhombic

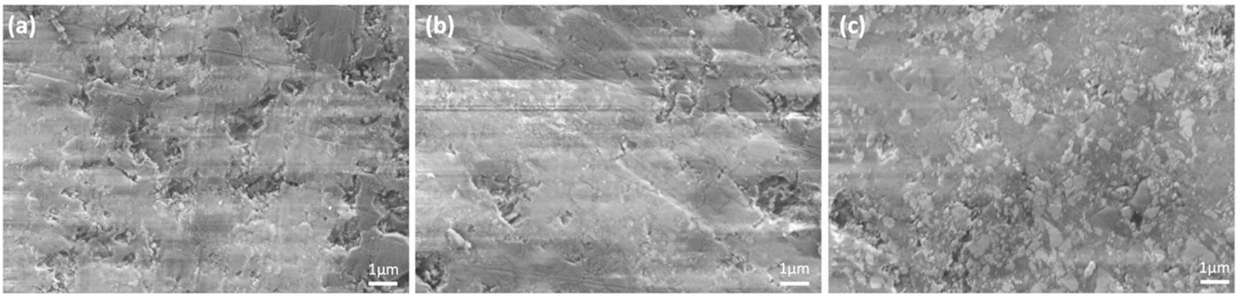

Figure S1. SEM images of sintered glass ceramics at 20.00 KX a) 30Al-O, b) 40Al-O, c)50Al-O.

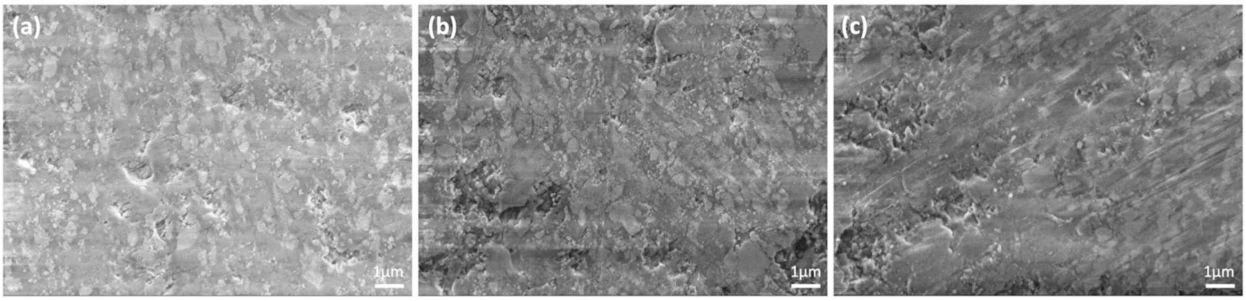

Figure S2. SEM images of sintered glass ceramics at 20.00 KX a) 30Zr-Ar, b) 40Zr-Ar, c)50Zr-Ar.

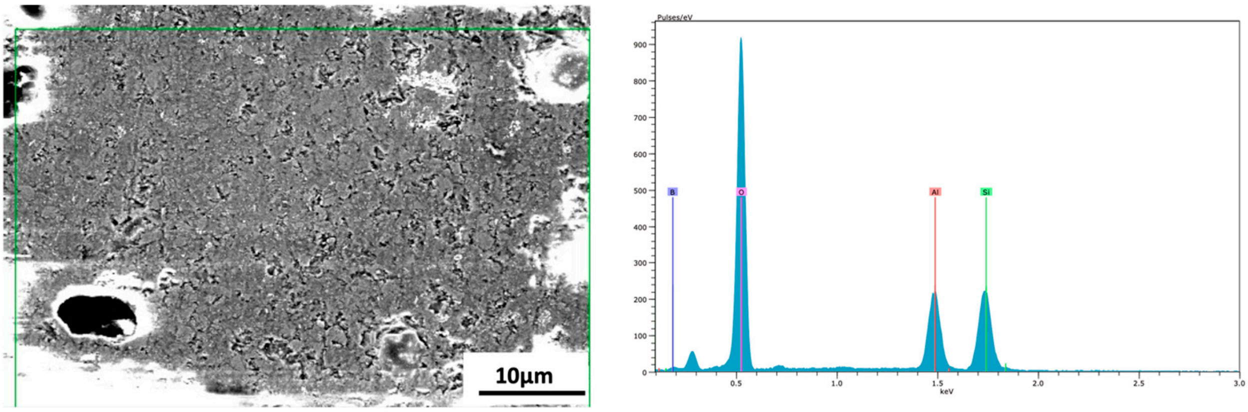

Figure S3. EDS analysis of the 30Al-O sample.

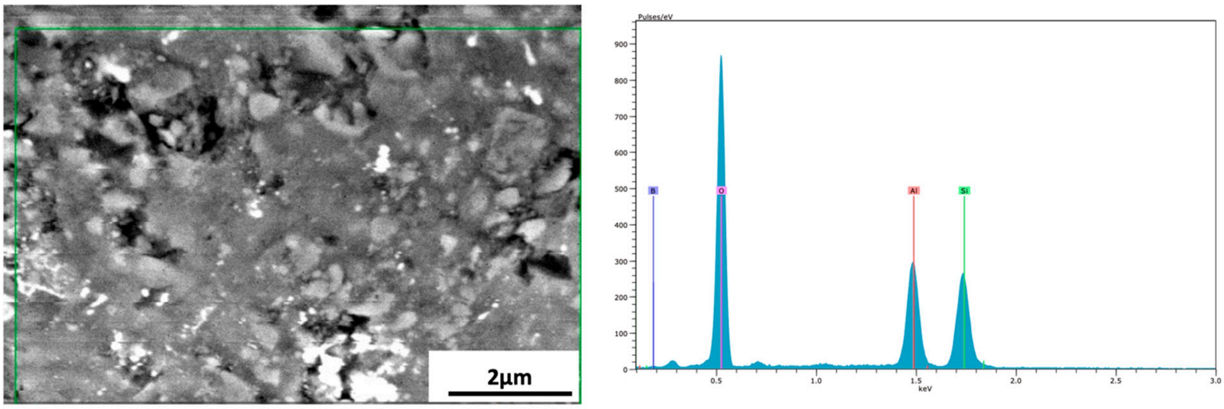

Figure S4. EDS analysis of the 40Al-O sample.

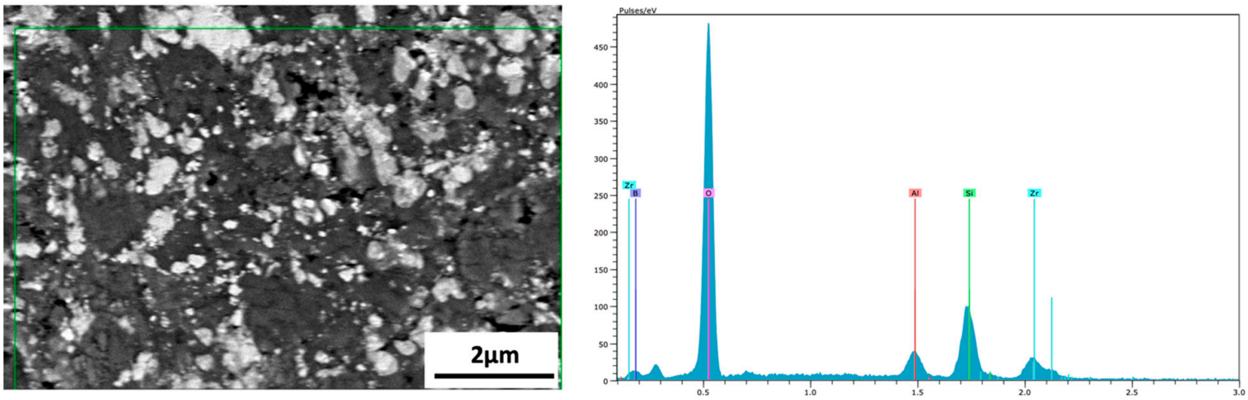

Figure S5. EDS analysis of the 30Zr-Ar sample.

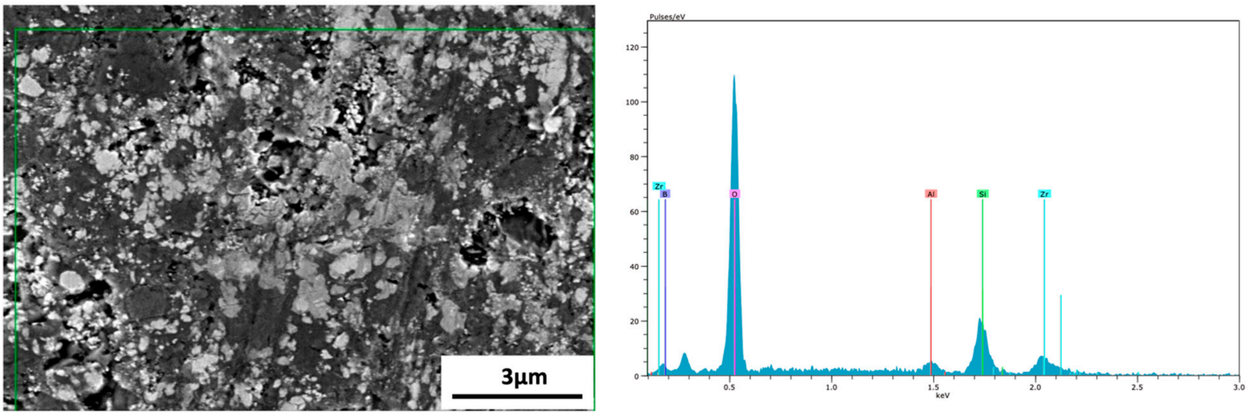

Figure S6. EDS analysis of the 40Zr-Ar sample.

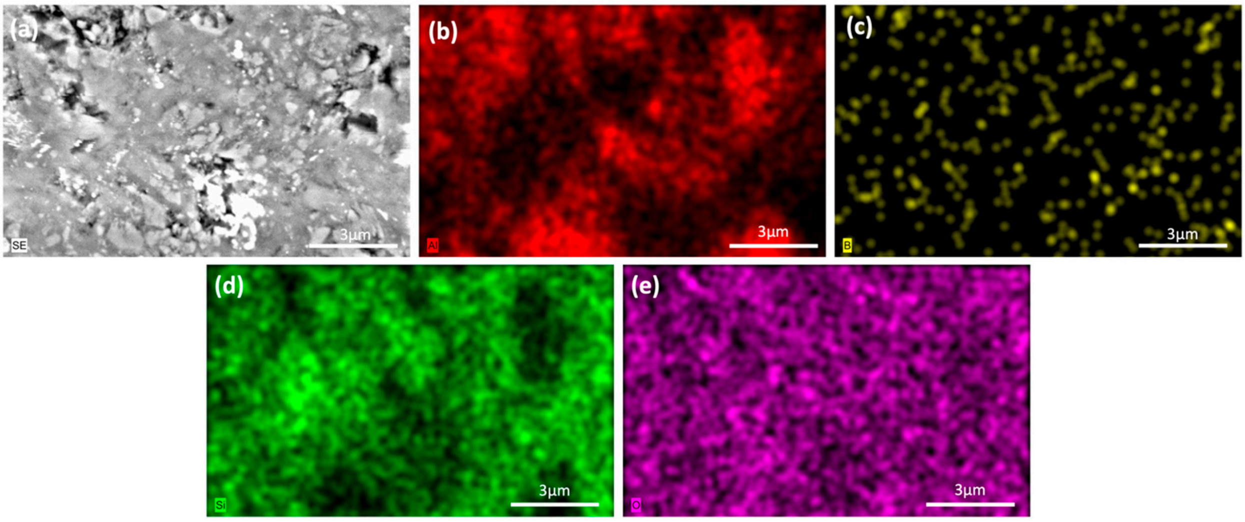

Figure S7. SEM/EDX analyses of the 40Al-O sample (a) SEM image, (b) EDX mapping of Al, (c) EDX mapping of B, (d) EDX mapping of Si, (e) EDX mapping of O.

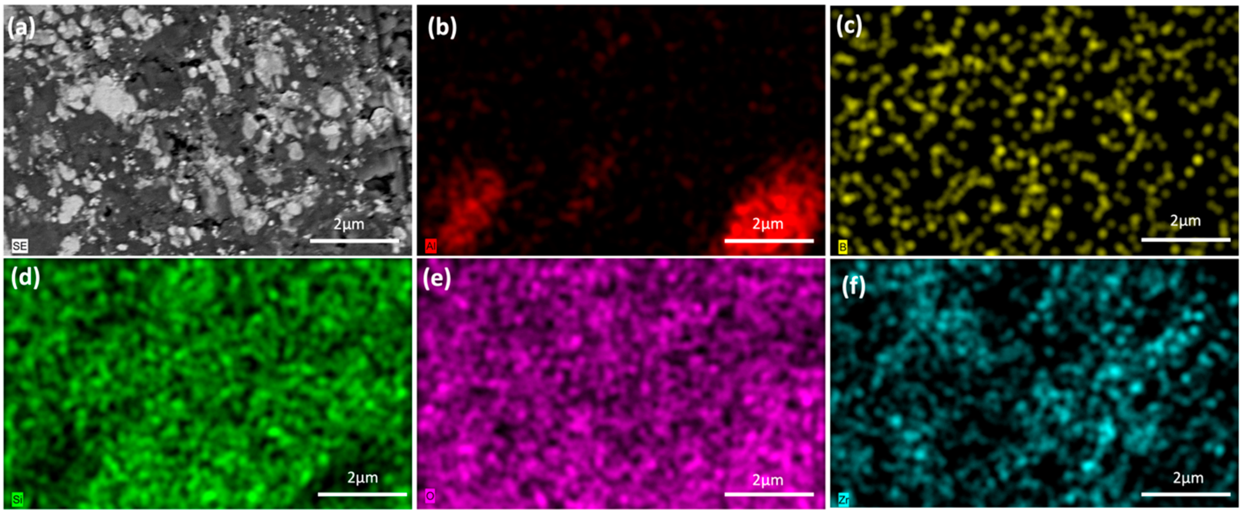

Figure S8. SEM/EDX analyses of the 30Zr-Ar sample (a) SEM image, (b) EDX mapping of Al, (c) EDX mapping of B, (d) EDX mapping of Si, (e) EDX mapping of O, (f) EDX mapping of Zr.
